# Resilience during lockdown: a longitudinal study investigating changes in behaviour and attitudes among older females during COVID-19 lockdown in the UK

**DOI:** 10.1186/s12889-024-19480-z

**Published:** 2024-07-23

**Authors:** Lan Li, Ava Sullivan, Anwar Musah, Katerina Stavrianaki, Caroline E. Wood, Philip Baker, Patty Kostkova

**Affiliations:** 1 UCL Centre for Digital Public Health in Emergencies (dPHE), Department for Risk and Disaster Reduction, University College London, London, WC1E 6BT UK; 2 EcoHealth Alliance, New York, USA; 3 Department of Geography, University College London, London, UK; 4 Department of Statistical Science, University College London, London, UK; 5 Crisis Response, British Red Cross, London, UK

**Keywords:** Resilience, Lockdown, Adaptation, Older female, Behaviour, Attitude

## Abstract

In order to slow the spread of COVID-19, on March 23, 2020, a strict lockdown was implemented in the UK. This was followed by alternating periods of less restrictive lockdowns until most public health restrictions were lifted in the summer of 2021. While these measures were necessary, they significantly affected people’s daily activities, lifestyles, and mental well-being.

This paper presents a longitudinal research study that focused on females aged 55 + in the UK, aiming to understand how COVID-19 and the subsequent 15-month period of lockdowns affected their lifestyles and emotions. The study collected data through online surveys, where respondents reported the frequency and mode of access to activities, and their positive and negative emotional experiences during distinct study phases that encompassed both lockdown and less strict periods.

In contrast to previous studies highlighting vulnerabilities for females and the elderly during lockdowns, this research found that the studied group maintained an overall positive outlook throughout the study period and successfully adapted to the lockdowns by increasing their engagement in certain activities, in particular, activities like ‘getting active’. In addition, our findings indicate rapid adaptive behaviour change towards many online activities, such as cultural activities. However, the study also revealed negative emotions and a decrease in some activities during the second lockdown in 2020 and the subsequent lockdowns in 2021, underscoring the challenges inherent in prolonged periods of confinement. In addition, the study found negative affect associated with some activities, including working and studying from home.

The findings provide valuable insights into how females aged over 55 coped with stressful circumstances, which can inform the development of resilient and mental health-conscious public health policies and responses in preparation for future pandemics or other hazards.

**Supplementary Information:**

The online version contains supplementary material available at 10.1186/s12889-024-19480-z.

## Introduction

In December 2019, SARS-CoV2 was first discovered in Wuhan, China, and spread globally ever since [1, 2]. Taking into account the scope and scale of the disease spread across the world, the World Health Organization (WHO) declared COVID-19 a pandemic on 11 Mar 2020 [3]. As of March 2020, there were over 100,000 confirmed cases and more than 4,000 deaths worldwide. By the end of 2020, global case numbers had surpassed 75 million, with nearly 1.6 million deaths reported [4]. The alarming case numbers and death rates have led much of the world to implement strict national lockdowns, stay-at-home orders, and other public health mandates to reduce the spread of the virus [5].

While it has been shown that lockdowns are effective in slowing the spread of COVID-19, lockdowns may also drastically alter people’s lives, as well as effect multiple aspects of the global, public, and private economy and way of life [6–8]. COVID-19 lockdowns were put in place to slow the spread of the virus across the world but also affected mental health and how people perform their daily activities [9]. The strictness of lockdowns was intersected by additional periods of various movement restrictions and other public health measures [10]. The prolonged periods of isolation, uncertainty, and fear associated with the pandemic have been shown to have profound effects on mental health and wellbeing [11]. Studies have reported increased levels of anxiety, depression, and stress among various populations during lockdowns [12]. The disruption of routine, limited social interactions, and economic uncertainties exacerbated these mental health issues [13]. Vulnerable groups, such as the elderly, were particularly affected [14]. Understanding the linkage between mental health and well-being during the pandemic is crucial for developing effective public health responses and support systems.

It is hard to conceptualise a single economy, population or sector which was not transformed in some part by COVID-19. Despite this, the effects that COVID-19 lockdowns had on individual populations or demographics were not felt equally across populations and demographics [15]. For example, some livelihoods that rely on in-person contact were unable to shift to remote/virtual working (e.g., barbers, cleaners), while others experienced widespread redundancies and layoffs due to shifts in consumer behaviour in light of shut-downs and travel bans (e.g., flight attendants). Despite the overall expectation for people to work from home, a study shows that only about 25% of U.S workers held jobs that could be adjusted to be performed in a fully remote setting [16], while only 57.2% of workers in London were able to work from home during the lockdown [17]. In a 2020 study, it was found that over one-third of UK adults who were employed full-time were worried about losing their jobs, while one-fifth of unemployed adults in the survey said they had had suicidal thoughts and feelings over the two-week study period [18]. Further, many recent studies have shown that females were more vulnerable to the COVID-19-related policy change, and tend to be disproportionately affected by the lockdown due to the existing gender inequality [10, 19–22]. The challenges faced by women include increased caregiving responsibilities, higher rates of job loss, and greater exposure to domestic violence [23]. These factors have contributed to higher levels of stress, anxiety, and depression among women compared to men [23]. The disproportionate burden of unpaid care work and the interruption of social support networks have further exacerbated mental health issues for many women [24]. These mental health changes experienced by people with different employment statuses, age groups, and demographic areas are critical to understanding how public health measures and interventions should be deployed and targeted.

We know from other types of disasters, like floods and hurricanes, that public health and safety mandates require consideration of the special needs and abilities of individual populations [25]. For example, during hurricane evacuation orders, disaster response specialists have identified particularly vulnerable groups which require specific and targeted evacuation plans, such as the elderly, those who are medically dependent on devices with power requirements (like refrigeration for insulin or a dialysis machine), pregnant women, or people with pets [26]. The considerations of specific differences experienced by people facing lockdowns during pandemics need to be considered to maximize public health impacts and minimize unintended consequences of lockdowns.

There is a paucity of knowledge about the differences between groups of people experiencing the challenges during the COVID-19 lockdowns. While there is a plurality in the exact definition across different disciplines, the concept of vulnerability is used in the field of disaster risk reduction in order to understand the conditions which increase the susceptibility of an individual, community or system to the impact of hazards [27]. The concept of resilience within the disaster risk reduction discipline refers to ‘the ability of groups or communities to cope with external stresses and disturbances as a result of social, political, and environmental change’ [28]. We may expect that certain demographic groups experience both vulnerability and resilience to mental health challenges during COVID-19 lockdowns. Vulnerability and resilience are not opposing concepts; a group can be simultaneously vulnerable to hazards while also possessing resilience to the impact of a hazard [30]. Resilience Theory offers a framework for understanding how demographic groups dealt with the challenges presented by the COVID-19 lockdowns and elucidates the underlying mechanisms [29]. For this analysis, we will focus on older females in the UK.

Females aged 55 and above represent a unique demographic that combines several factors of vulnerability and resilience. Age is a demonstrated risk factor for severe illness during COVID-19 infection [31]. Individuals aged 55 and above are often qualified for senior living communities and related services and are typically categorized as older adults, particularly concerning health and social care needs [32, 33]. As such, in this study, individuals aged over 55 are identified as a group susceptible to the impact of COVID-19, encompassing challenges related to physical health and mental well-being, including experiences of stress and fear. Additionally, this demographic has limitations related to information use and information technology skills, which is particularly prescient during the COVID lockdowns where activities like online shopping and virtual conferencing were crucial [34–36]. However, it is essential to acknowledge that individuals aged 55–60, may still be actively involved in responsibilities like parenting, supervising remote learning during lockdowns, or caring for elderly family members. The at-risk nature of this demographic is amplified by other risk factors, such as residing in nursing homes, cohabitating with individuals employed in high-risk occupations, or having pre-existing health conditions like asthma, obesity, or diabetes [36–38]. Similar to age, gender is also a key consideration when understanding vulnerability or resilience to hazards [39, 40]. There is evidence that there are gender differences not only between the morbidity and mortality of COVID-19 but also the attitudes and behaviours associated with COVID-19 [41]. Thus, females aged over 55 were selected as the targeted population in this study to capture a group that is particularly vulnerable yet also capable of demonstrating resilience through adaptive behaviours.

Given these considerations, this study aims to explore how females aged 55 and above in the UK cope with the challenges posed by the COVID-19 pandemic and subsequent lockdowns. By focusing on this demographic, the research seeks to provide valuable insights into their behavioural adaptations, emotional resilience, and overall well-being during this unprecedented crisis. This information allows scientists, policymakers, and the public to create specific public health measures which consider the complexities and diversity of the lived experience during a disaster.

## Methods

The present research comprises a segment of a broader study that explores the impacts of the COVID-19 pandemic on the behaviours, lifestyles, and emotional states of UK inhabitants over the course of more than 15 months (April 2020 to July 2021) [4]. The study was conducted in six phases which were deployed to correspond with key COVID policy changes (e.g.: lockdowns and reversals of lockdowns), spanning April 2020 to August 2021. Additional details on these study phases can be found in a previous manuscript [7]. We utilized online surveys to collect data from participants recruited through a snowball sampling method facilitated by online advertisements. The current study specifically focuses on females aged 55 years and older to elucidate their experiences and adaptability to COVID-19 restrictions, particularly in relation to their vulnerability and resilience. The longitudinal design allowed for repeated measures from the same individuals across different phases, providing a comprehensive view of changes over time.

### Measures and questionnaire design

**Fig. 1:**
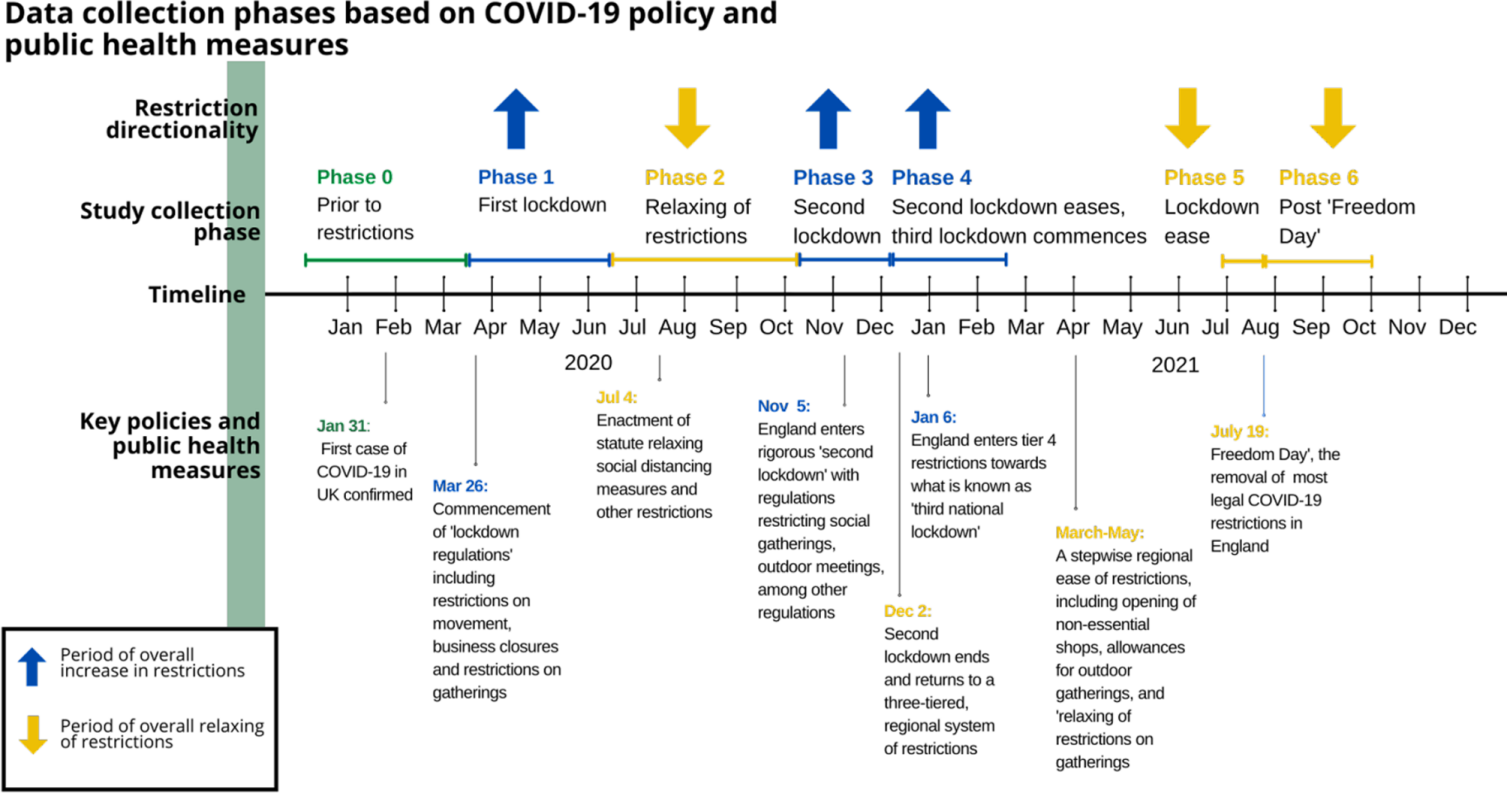
Data collection phases based on COVID-19 policy and public health measures

The purpose of the questionnaire is to assess changes in individual behaviours in response to the six phases of the COVID-19 pandemic, which are defined by Fig. 1 to represent periods of strict lockdown and less restrictive phases. The original questionnaire consisted of three sections with a total of 48 items. The first section assesses the frequency and mode (online or in-person) of activities performed at the time of the survey, measuring 16 activity types using categories defined by the UK National Time Use Survey [30]. Each activity is measured by two questions, assessing frequency (“How often do you currently engage in the following activities.”) and mode (“How has the mode of engaging in each activity changed compared to before COVID-19?”). Frequency is measured by six ordinal options ranging from “never” to “more than once per day,” and mode is measured by four options, including “nothing changed” (compared to the way before the pandemic, which is assumed as fully in-person), “some online and some in-person,” “fully online,” and “stopped.” The second section of the questionnaire includes demographic questions to measure participants’ background, such as age, gender, education, employment status, household number, and household characteristics.

The third section assesses emotional status using the Positive and Negative Affect Schedule (PANAS), a well-established psychometric tool used to measure positive and negative affect [31, 32]. The scale consists of 20 items, with 10 items measuring positive affect (PA) and 10 items measuring negative affect (NA). Respondents rate each item on a 5-point Likert scale ranging from 1 (very slightly or not at all) to 5 (extremely). The selection of the PANAS scale over other competing measurement tools was based on its proven reliability and validity, as well as its concise format, which reduces respondent burden while providing comprehensive data on both positive and negative affect. Compared to other scales, such as the Profile of Mood States (POMS) or the Beck Depression Inventory (BDI), the PANAS offers a balanced assessment of both positive and negative emotions, which is crucial for a holistic understanding of emotional well-being during the pandemic [42–43]. The details of the PANAS analysis for the entire population can be found in our previous publication [44].

While all three sections remained present across each questionnaire administration phase, each survey varied slightly to address changes across the public health situation. For instance, three vaccine-related questions were added to the questionnaires at the beginning of phase four. A comprehensive version of the questionnaire, including all questions across each survey version, is included in the S1 File.

### Recruitment and data collection

During a period of the COVID-19 pandemic spanning from April 2020 to July 2021, a comprehensive online survey was conducted to investigate the behavioral changes of individuals. The survey was divided into six distinct phases, with the first phase termed “phase 0”, involving the comparison of behavioral changes to the pre-pandemic baseline. The survey was advertised through various social media channels such as Facebook and mutual aid groups. Subsequently, 3,240 participants voluntarily provided their email addresses and partook in the first survey. Using the email list provided by the initial cohort, a further five surveys were conducted within the same cohort, with the second, third, fourth, fifth, and sixth surveys conducted in May 2020 (*n* = 1,399), October 2020 (*n* = 856), December 2020 (*n* = 1,050), June 2021 (*n* = 1,298) and July 2021 (*n* = 1,036), respectively. For the third and fourth surveys, we recruited new participants through Facebook advertising in October 2020 (*n* = 1,762) and December 2020 (*n* = 143). All survey data was collected using SurveyMonkey.com.

### Data cleaning and analysis

The following analysis involved data analysis on a sample of individuals. Firstly, incomplete records were removed to ensure data integrity. Subsequently, sample participants were filtered by gender and age, retaining only females aged 55 and over for analysis. To gain insight into the frequency of activities performed, a frequency analysis was conducted on self-reported data. Given the nature of the data and deviations from normality, non-parametric tests were employed for the following data analysis.

Chi-squared goodness of fit tests were carried out phase-by-phase to explore whether there were significant differences in the mode and frequency of activities separately. This test was chosen due to its suitability for categorical data and its ability to compare observed frequencies to expected frequencies. Significant activities identified through these tests were then selected for further descriptive analysis. To visually assess the distribution of data, a grouped stacked bar plot was used to present the frequency of activity by phase and boxplots were employed to assess the overall PANAS scores by phase. To establish the relationship between the activity and PANAS, a series of boxplots was generated to gain an understanding of the overall data distribution for selecting the appropriate statistical tests. The frequency and mode of the activities were considered separately, given the differences in data types. Since the frequency variable is ordinal data (ranked from “never” to “more than once a day”) we dichotomized the data into numeric values ranging from 1 to 6.

To explore the relationship between the frequency of activities and the PANAS score, the Kendall correlation coefficient tau and p-value were calculated. This non-parametric correlation measure was chosen due to its robustness in handling ordinal data and its ability to provide meaningful correlation coefficients even with non-normal data. To examine mode change, whereby the activity was performed either online or in-person, only the ‘in-person’ and ‘online’ options were retained, while ‘some online, some in-person’ and ‘never’ were discarded. We then analysed how the mode of activity (online or in person) impacted mood, as represented by the PANAS score. The mode variable, being categorical data, required the use of two-sided Wilcoxon tests to determine the mode’s significant correlation with the PANAS score. The Wilcoxon test is appropriate for comparing two related samples or repeated measurements on a single sample to assess whether their population mean ranks differ. All analysis was conducted for each activity and each phase. Finally, the results were aggregated using heatmaps and grouped boxplots. A statistically significant test was defined if the p-value was less than 0.05. All statistical tests were carried out on the RStudio desktop version 2021.09.1 + 372 [45]. The data visualization was partially conducted on Tableau 2022.3.

## Results

### Sociodemographic characteristics

The survey reached 4,992 participants, 1,577 of whom were aged below 55 and 1,218 were male, resulting in 2,197 participants included in this study and contributing to 5,754 records in 6 phases. Among the included sample, 44.11% of the participants were older than 65 (*n* = 969). The majority of the participants had a high-level education with college or university degree (48.52%) or post-graduate degree (27.72%), were from a White ethnic background (98.82%), were retired (51.48%), and living in the south part of England at the time of the survey (Top 3 regions: South East, London, and South West). Table 1 displays the socio-demographic details.

**Table 1:** Socio-demographic details of the included participants

Demographic categories	Number (Percentage) (*N* = 2197)
**Age**	
55–64	1228(55.89%)
65+	969(44.11%)
**Employment**	
Employed	893(40.65%)
Not employed	173(7.87%)
Retired	1131(51.48%)
**Education**	
College or university	1066(48.52%)
Higher or secondary or further education	295(13.43%)
Less than secondary	18(0.82%)
Post-graduate degree	609(27.72%)
Secondary up to 16 years	209(9.51%)
**Household number**	
Alone	526(23.94%)
2	1250(56.90%)
3	257(11.70%)
4	115(5.23%)
5 or more	39(1.78%)
**Ethnicity**	
White	2171(98.82%)
Mixed	19(0.86%)
Asian	6(0.27%)
African and Black	1(0.05%)
**Garden**	
No	49(2.23%)
Communal garden	53(2.41%)
Private garden	1217(55.39%)

### Change in frequency of doing various activities

**Fig. 2:**
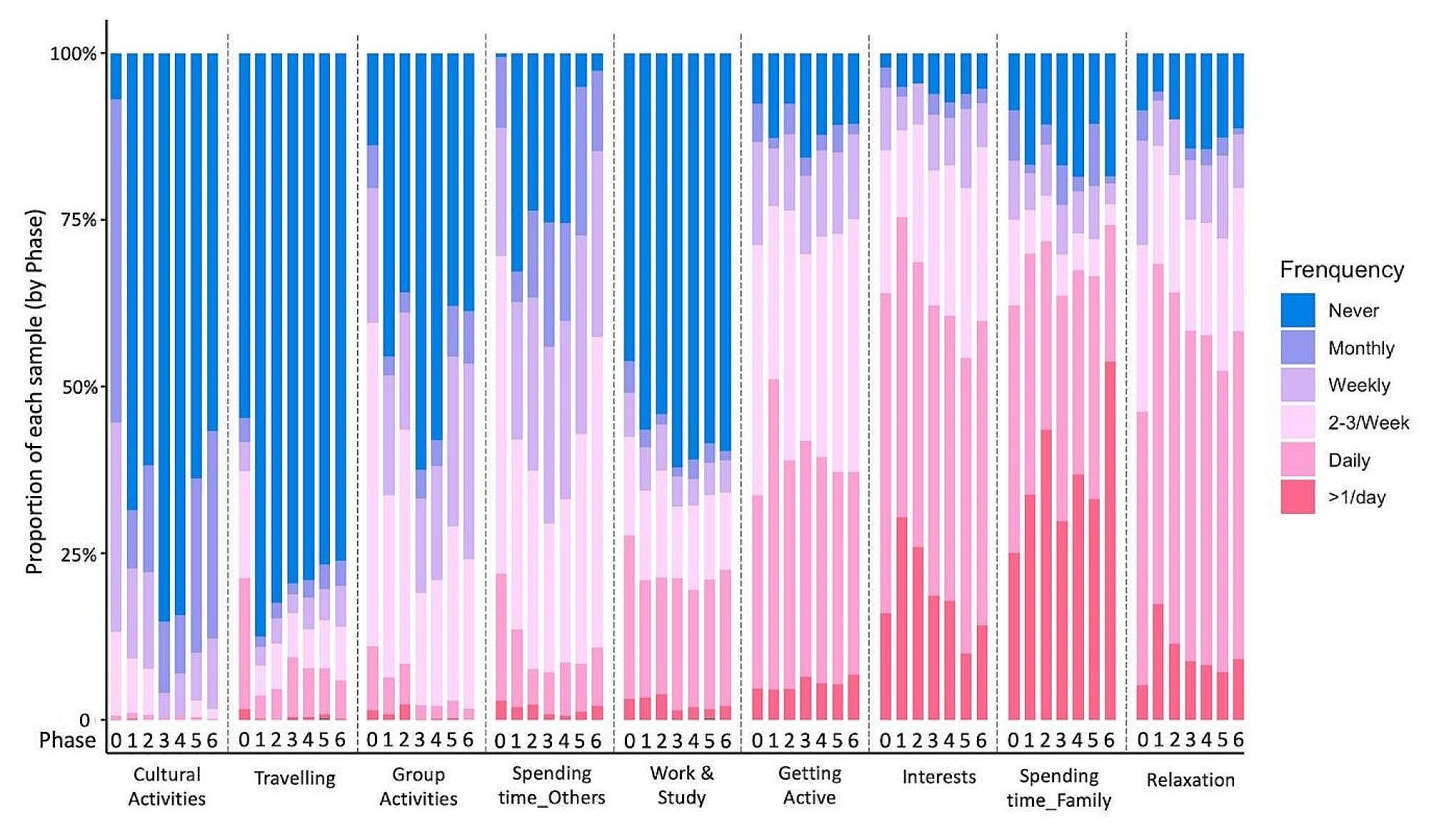
Stacked bar chart showing the frequency changes of activities across 7 phases (filtered by Chi-square p-value below 0.05, which indicates significant change in this activity throughout the study period)

To understand the change in frequency people accessed and engaged in different activities across the different phases of lockdown, we analysed the data of frequency (6 Likert-scale options from “never” to “more than once per day”) for each activity. The Chi-square goodness of fit test was first used to test the significance of change for frequency changes in each activity, and the activities with significant frequency change (p-value below 0.05 by Chi-square test) are presented in Fig. 2.

As can be seen from Fig. 2, cultural activities, travelling, group activities, and spending time with others were significantly reduced from phase 0 to phase 1, indicating that the participants decreased engagement with these activities once the pandemic reached the UK in April to June 2020. Although some frequency of activities were captured from phase 4 to 6 (January to August 2021), people tended to carry out these activities much less frequently than before the pandemic, even in phase 6, when nearly all the public health measures were abolished (phase 0 vs. phase 6). This is particularly true for cultural activities, travelling, and group activities, where the reported frequency of these activities significantly decreased. In contrast, some activities, like ‘getting active’, ‘pursuing interests’, ‘spending time with family’ and ‘relaxation activities’, increased from phase 0 to phase 1. This shows that people are more frequently engaging in these activities during the first lockdown than before the pandemic. However, in most cases, this increase declined from phase 1 to phase 4, with the exception of spending time with family. Although some activities increased again between phases 5 to 6, the frequency of people doing these activities is generally similar to those prior to the pandemic.

### Change in positive and negative feelings throughout the pandemic

**Fig. 3:**
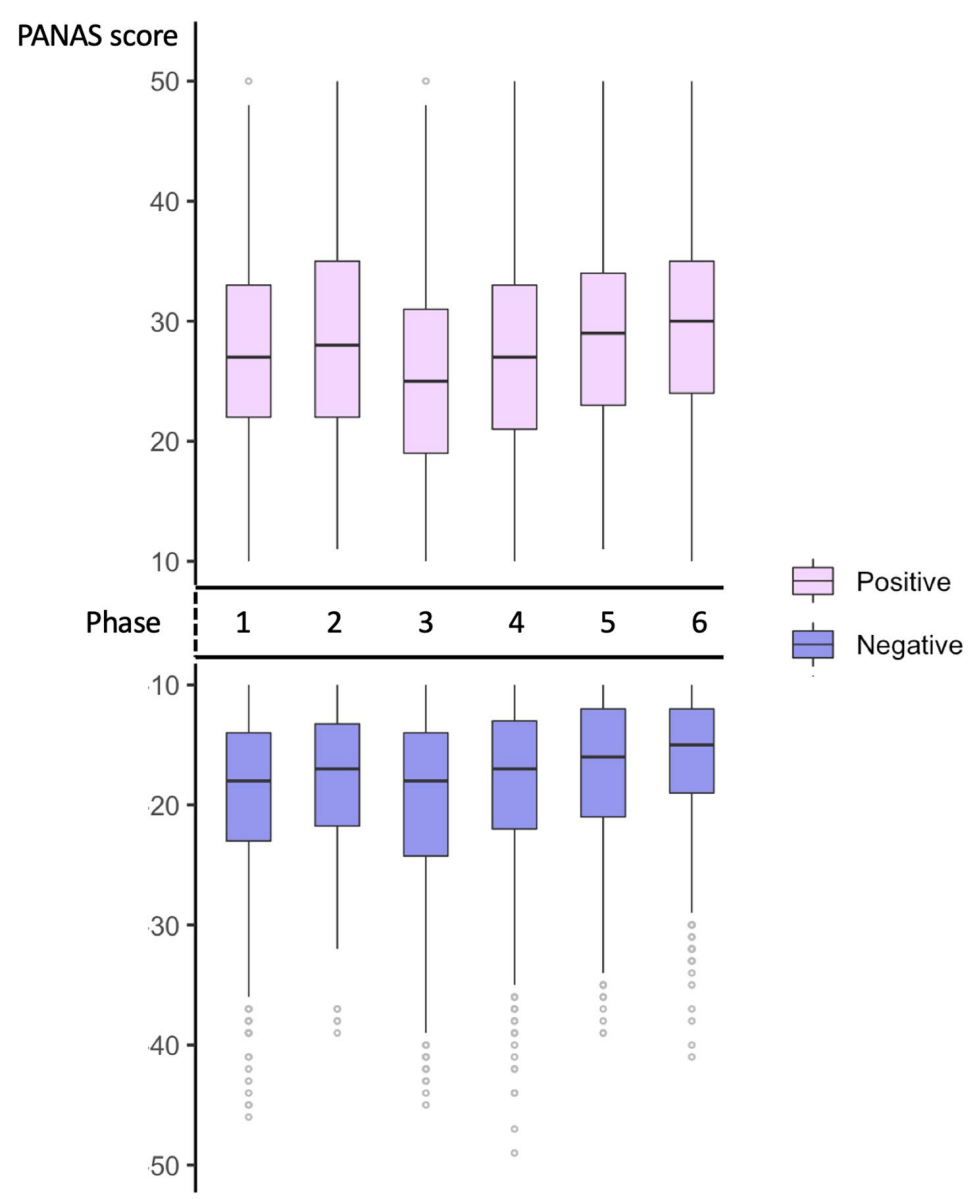
The boxplot showing the overall positive affect scores (PA) and negative affect scores (NA) throughout the pandemic (reading upwards for the PA and downwards for the NA scores)

The scores for positive and negative affect throughout the 6 phases are illustrated in Fig. 3. In order to read the results of the boxplots, the PANAS score is illustrated on a scale from 0 to 50, reading upwards for the positive affect scores (PA) and downwards for the negative affect scores (NA). As such, the figure can be read by identifying the top-most positive boxes as the most positive feelings, whereas the bottom-most boxplots represent the most negative feelings, while high scores represent high positivity and high negativity.

Overall, this group reported an overall high positive affect and lower negative affect across all 6 study phases, indicating a generally positive attitude across the full study period. From the graph, we can see that participants reported higher negative emotions in phase 3, with both low positive affect scores and high negative affect scores. Conversely, the group reported more positive emotions in phase 6. Overall, when higher positive affect scores were reported, lower negative affect scores were also reported, as was the case in phases 1 to 2 (affect reported as more positive and less negative), phases 2 to 3 (scores showing higher negative scores and lower positive scores), and in phase 3–6 (scores becoming slightly less negative and slightly more positive).

### Activities during lockdown impact positive and negative emotions

**Fig. 4:**
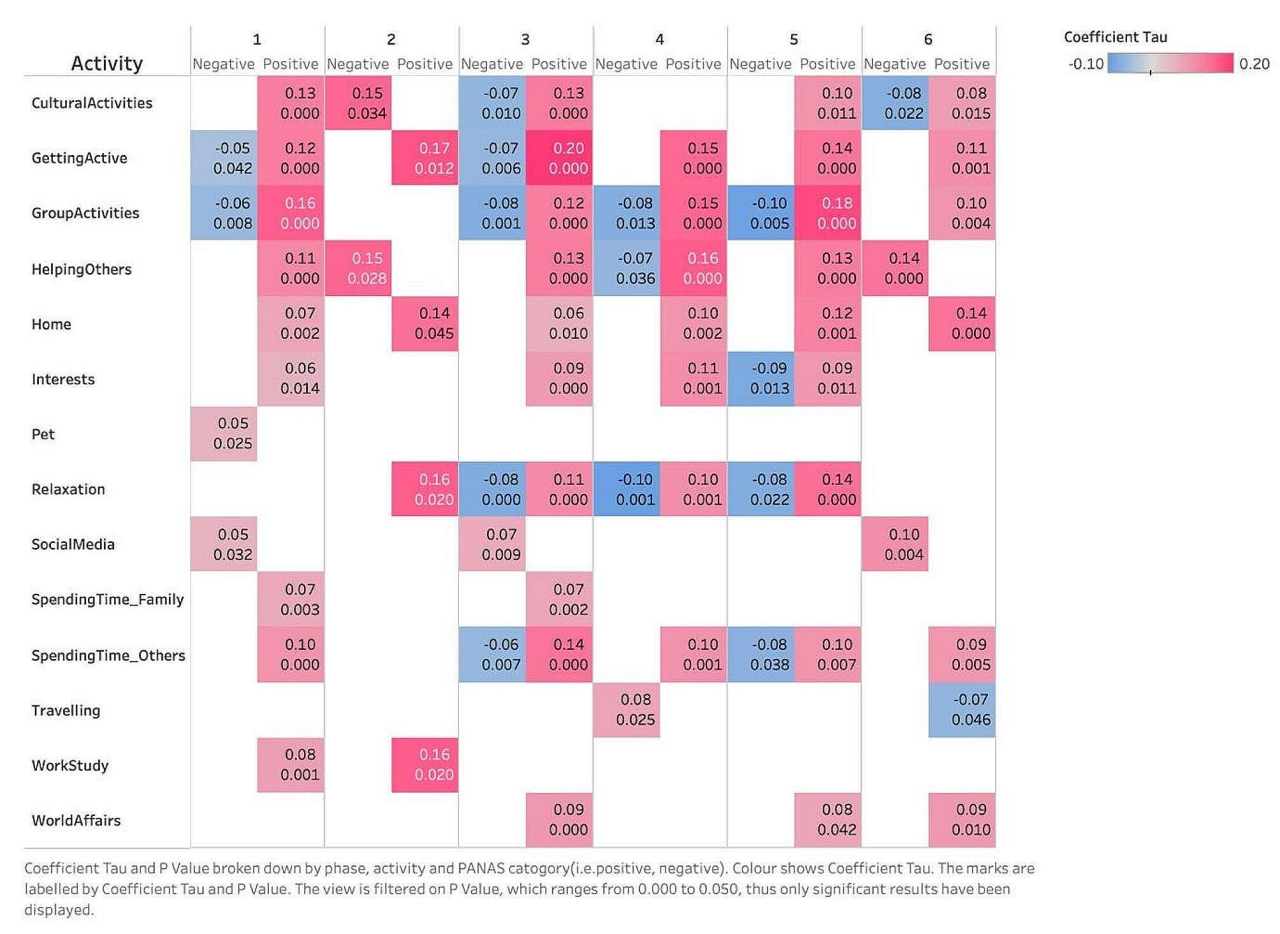
The heatmap of correlation between PANAS score and the frequency of activity. (Calculated by Kendall’s correlation test. Coefficient Tau and p-value broken down by phase, activity and PANAS category (positive and negative). Colour shows Coefficient Tau. The marks are labelled by Coefficient Tau (up) and p-value (down) in each cell. The view is filtered on p-value, ranging from 0.000 to 0.050; thus, only the pairs that have significant correlation have been displayed.)

Below is an analysis of the reported positive and negative affect scores as they relate to different self-reported activities during the COVID-19 lockdowns. The correlations between the frequency of doing each activity and the PANAS scores were tested using the Kendall correlation tests for each activity and PA and NA scores separately. The significant results are reported in Fig. 4. The complete results can be found in Supplementary Material 2 (S2 Figure).

As illustrated in Fig. 4, the coefficient value (Tau, in the range of [-1,1]) is between − 0.10 and 0.20, indicating there are modest correlations between the frequency of activities and PANAS scores. While the coefficient was low, results are still determined to be reportable, as a correlation was found through this analysis. It can be seen that positive affect scores are mostly positively correlated with the frequencies of activities, while negative affect scores are mostly negatively correlated, which indicates that engaging in more frequent activities is largely associated with more positive feelings. However, this is not the case for two activities: social media and travelling. The correlation between the frequency of using social media and negative affect scores in phases 1, 3 and 6 remains positive, which indicates more frequent social media use is associated with more negative feelings. Interestingly, another activity measured through the study, that of engaging in ‘world affairs’ (which encompasses watching or reading news media), shows the reverse effect. Females in the study who reported following world affairs through watching or reading the news generally reported higher positive affect scores. Females in the study who reported frequent travel in phase 6 have significantly lower positive affect scores, indicating that this activity was related to less positive feelings.

**Fig. 5:**
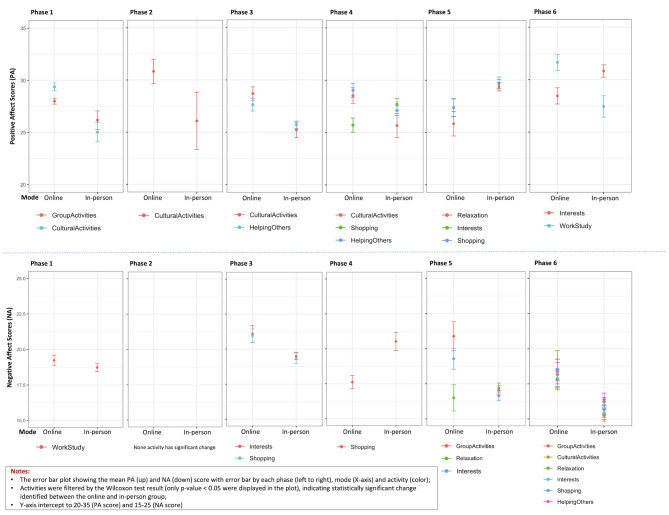
Error bar charts comparing the mean of PA score (up) and NA score (down) with standard errors by modes (x-axis), activities (colour) and phases (left to right) filtered by Wilcoxon test p-value (< 0.05)

A Wilcoxon signed-rank test (two-sided) was used to explore the relationship between participants’ mode of access to activities (online or in-person) and their reported PANAS scores. For each activity, the positive affect and negative affect scores were separated into two groups: in-person and online, and then a series of Wilcoxon tests were conducted to test the difference of PA and NA scores of the two mode groups. Only significant results, with a p-value below 0.05, were reported in Fig. 5 (See S3 Table for full results).

Notably, different and limited activities were captured with significant Wilcoxon test results between PA and NA scores. From the PA score, we can see those accessing cultural activities online have significantly high PA scores from phases 1 to 4. Similar results were found among those accessing online group activities in phase 1, and online activities related to helping others in phase 4. In contrast, the activities done in person in phases 5 and 6 tended towards higher positive affect scores. Exceptions to this trend were found in those working and studying during phase 6, where those who were performing these activities online had significantly higher PA scores. Different patterns were displayed in the results of the NA scores. From phase 1 to phase 6, the significant activities presented in the charts illustrate higher negative affect scores among the online cohort than those among the in-person cohort, indicating that the females in the study are reporting more negative feelings when accessing the same activities online.

## Discussion

Our study aimed to explore the emotional and behavioral adaptations of females aged 55 and over in the UK during the COVID-19 lockdowns, focusing on how these changes relate to their positive and negative emotional experiences as measured by the Positive and Negative Affect Schedule (PANAS), and the changes in frequency and mode of access (online or in-person).

Overall, this group reported a net positive affect score and net negative affect scores across the duration of the study period. While this group was often reported as disproportionally affected by COVID-19 [10, 19–22], in this study we in fact demonstrate that this group has an overall positive outlook and the ability to adapt to the lockdown’s demands. This result is notable given the inherent challenges represented by the COVID-19 pandemic and resulting lockdowns. The overall positivity of this group may be a result of the unique demographics of our sample; they were largely spared from livelihood loss associated with younger people, as well as many having access to resources such as a private garden and internet.

As a result of changing public health policies, nearly all kinds of activities and lifestyles were affected throughout the pandemic [7, 46]. Our findings show that females over 55 adapted to the lockdown by increasing certain activities, such as relaxing, getting active and exploring their interests. However, the frequency of other activities, such as social activities (e.g. cultural activities, group activities, spending time with others), decreased during the first lockdown, likely in accordance with the public health policies that limited such behaviours. These same adaptive activities increased from phase 3 to 6, indicating that this population resumed activities since the second lockdown, showing some ability for resilience post-COVID and readiness to pursue the ‘new normal’.

Compared to the first lockdown, the participants had more negative feelings in the second lockdown, which may demonstrate lockdown fatigue or an inability to change behaviour as readily during the repeated lockdown measures [47]. A similar decreasing frequency pattern across the first and second lockdowns was found in activities related to pursuing interests and getting active. The study found that certain activities, such as accessing cultural activities, travelling, group activities, and spending time with others, never resumed the same frequency from that of the beginning of the pandemic. This may indicate the lasting impact that COVID-19 has had on our behaviours, despite the lack of restrictions in place post-‘Freedom Day’, which was consistent with previous studies [46, 48, 49].

The analysis of specific activities and affect scores indicated that there is a relationship between women performing certain activities and their feelings during the lockdowns. Engagement in physical activities emerged as a significant factor in maintaining positive affect among the participants. Participants who reported an increase in getting active during phases 1 to 6 reported more positive feelings. The correlation analysis demonstrated that those who frequently participated in physical activities during lockdown phases reported significantly higher PA scores, particularly during the initial lockdown period (Phase 1).

The study found a positive correlation between increased social media use and higher negative affect scores across several phases. This suggests that while social media served as a means to stay connected, it also contributed to negative emotions, possibly due to exposure to distressing news and the lack of face-to-face interaction. More frequent social media use was associated with more negative feelings, whereas the same is not the case for accessing world affairs through new media. These findings are mostly consistent with previous similar research [50–52].

Results from this study also show that many females found access to activities online, such as cultural activities, were related to higher positivity. This may indicate that for this population, there is respite in the provision of online or virtual activities during the lockdown, which is a very interesting outcome contradicting expectations and other studies [14, 35]. However, for the same population, working or studying online was related to higher reports of negative affect. Working and studying from home presented a critical area of concern. Participants who engaged in these activities online reported higher negative affect scores, reflecting the challenges and stress associated with adapting to remote work and education environments. This finding is important to understand this population’s challenges when faced with working or studying from home.

The findings can be better understood through the lens of Resilience Theory. The observed increase in activities like online engagement, relaxation, and family time can be seen as adaptive strategies that participants employed to maintain their well-being. Resilience Theory posits that such adaptive behaviours are critical for mitigating the negative impacts of stressors [53]. The overall positive affect and ability to adapt observed in this study suggest that these females possessed resilience factors that helped them navigate the challenges of the lockdowns. Furthermore, the decline in negative emotions and the maintenance of positive activities during the second lockdown highlight the dynamic nature of resilience. Resilience is not a static trait but a process that evolves with changing circumstances [54]. The initial lockdown may have provided a learning experience, equipping females with strategies and coping mechanisms that they utilized in subsequent lockdowns. The significant correlation between positive affect and engagement in activities like exercise and cultural activities highlights the role of resilience-enhancing behaviours. Conversely, the association of negative affect with increased social media use may indicate that not all coping strategies are beneficial, highlighting the complexity of resilience.

The findings of this analysis have significant implications for public health policy, particularly in designing interventions and support mechanisms for older females during pandemics or other public health emergencies which require lockdowns. The analysis underscores the need for policies that address the specific vulnerabilities and resilience factors of older females, a demographic that has shown both significant adaptability and susceptibility to mental health challenges during prolonged lockdowns. We can leverage much of the information from this study into the tailored strategy for higher mental health outcomes during the next hazard resulting in lockdown.

It is notable that this population was able to change behaviour and maintain positive affect scores during the first lockdown period but reported more negative affect and less increase in beneficial activities during the second lockdown period. The ability of people to stay resilient during repeated lockdowns represents an extreme challenge and one that policymakers need to understand fully to better prepare for future pandemics. Our findings show the need for increased mental health support for our study population as the number of lockdowns increase over time. While our study population’s overall need is low (general positive affect across the period), the negative affect increases over subsequent lockdown periods.

Additionally, the findings related to the positive impact of pleasurable online activities, such as cultural offerings and the ability to help others, increase positive affect during trying times. A policy recommendation surrounding this finding may include supporting electronic literacy amongst older adults and females in order to ensure access to online offering are utilized during lockdown.

Another policy implication within our findings is related to working and studying from home as a source of negative emotions. Sensitivity to such realities should be taken into consideration by employers, teachers, and society at large, if and when lockdowns are necessary in the future. These results show that it is not just the ability to access online alternatives to activities that alleviate the stress of a lockdown situation, but rather, that in the case of certain activities, they can provide a positive outlet, whereas in other cases, it can be a source of anxiety, disconnection, or isolation.

### Strengths and limitations

This study has strengths in sample size and study design. With a relatively large sample, it included repeated responses across the UK and had relatively high proportions of older respondents (aged over 55). This study sheds a new light on adaptability and types of activities which keep older females in a more positive mood during the difficult period of lockdowns.

An obvious limitation of this study is the sample demographics. As described in the results, the participants were mostly highly educated, predominantly from a White ethnic background, and living in more affluent areas. This might be explained by the limitation of online surveys, which can only capture people with internet access, high connectivity, and e-literacy. Moreover, it is essential to acknowledge that the age categorization (55+) might oversimplify the diverse experiences within this group. Thus, it is imperative to refine our understanding of vulnerability within this age group, recognizing the variability in life situations of this diverse group.

Additionally, the reliance on self-reported data through online surveys introduces potential biases, such as social desirability bias and recall bias, which could affect the accuracy of the reported behaviours and emotional states. The study’s longitudinal design, while valuable, also presents challenges related to participant attrition over time, which can impact the consistency and reliability of the data collected across different phases.

Future studies should investigate this topic in a wider population, especially in people from lower educational backgrounds and those from minority ethnic groups, some of the worst affected groups by COVID-19 [55, 56]. Incorporating a mixed-methods approach, including qualitative studies such as telephone interviews, can provide deeper insights into the associations between activities and emotional well-being. Moreover, employing strategies to improve participant diversity and retention, such as targeted recruitment and follow-up efforts, could enhance the representativeness and robustness of the findings. Furthermore, our result shows only a weak correlation (< 0.3) between the way and frequency of people doing activities and their feelings. However, qualitative studies such as telephone interviews can be carried out to explore the associations further.

## Conclusion

Given the increased risk of severe disease from COVID-19 experienced by the older population, as well as the behavioral changes demanded during the pandemic, the target group analysed in this study (older females) were expected to be vulnerable to mental health challenges during the COVID-19 lockdowns in the UK. However, this in-depth study found this group to be overwhelmingly positive during this time period and measured an ability to remain positive throughout its ups and downs. Through the study, we also found a particular ability to change behaviour during the first pandemic lockdown, including increasing online activities that give pleasure, such as accessing cultural activities and getting active. Despite this, there remain challenges to remaining positive during the pandemic for this group. This study found a decrease in the frequency of many activities, as well as a decrease in positive affect during these same activities, during subsequent lockdown periods. In addition, the study found that certain activities were able to contribute to maintaining positive affect when accessed online, such as helping others and pursuing interests. However, positive affect when accessing online activities were not able to be maintained unilaterally. When faced with the challenges of working or studying online, this population experienced an increase in negative feelings. In addition, the study found a lasting effect on activities pursued overall over the life of the study; despite a complete lack of public health restrictions, a decrease in the overall frequency of activities was measured.

These lessons in behavior change and emotions for this group are valuable insights when it comes to creating a tenable and healthy public health response to future pandemics and other hazards. The results of this study should be considered when planning for future pandemics. The sample of UK females above 55 was largely white, educated, and had access to resources like private outdoor space. These conditions, which provide a baseline for the sample’s overall positivity during the pandemic, should be considered when applying the lessons learned. Despite this limitation, we can understand that virtual options for critical activities are not equal - access to passions, social time and leisure activities are positive investments for virtual offerings. However, when considering the sustainability of working or studying from home, an additional consideration for mental health counselling and sensitivity needs to be made. Likewise, we measured resilience to the first pandemic, in that participants were able to change behaviours towards activities that garnered positive affect but were less able to do so in the subsequent lockdowns.

This study not only provides empirical evidence of how older females coped during the COVID-19 lockdowns but also contributes to a deeper theoretical understanding of resilience in the face of public health crises. Future studies are required to understand this level of granularity in experience across diverse populations. Despite COVID-19 affecting the global population, socio-demographics play a large part in how the pandemic, and its resulting public health measures, affect individual communities, groups, and systems. The results of this study are an important contribution to the preparedness and planning of public health measures and support and coping mechanisms for older female groups for future pandemics.

### Electronic supplementary material

Below is the link to the electronic supplementary material.

Supplementary Material 1

Supplementary Material 2

Supplementary Material 3

## Data Availability

Data collected and analysed during the current study are available from the corresponding author upon reasonable request.
