# Supplementary material for: Resilience during lockdown: a longitudinal study investigating changes in behaviour and attitudes among older females during COVID-19 lockdown in the UK
Source: BMC Public Health. 2024 Jul 23;24:1967. doi: 10.1186/s12889-024-19480-z (PMC11267972; doi:10.1186/s12889-024-19480-z)
Supplement: Supplementary file 2 — Supplementary Material 2 [file 12889_2024_19480_MOESM2_ESM.pdf]

Activity		1		2		3		4		5		6	
		Negative	Positive	Negative	Positive	Negative	Positive	Negative	Positive	Negative	Positive	Negative	Positive
CulturalActivities	Coefficient Tau	-0.016	0.126	0.153	-0.028	-0.066	0.128	-0.054	0.018	-0.045	0.096	-0.080	0.084
	P Value	0.515	0.000	0.034	0.693	0.010	0.000	0.116	0.591	0.243	0.011	0.022	0.015
GettingActive	Coefficient Tau	-0.048	0.125	0.070	0.174	-0.066	0.200	-0.039	0.152	-0.057	0.138	0.059	0.105
	P Value	0.042	0.000	0.317	0.012	0.006	0.000	0.218	0.000	0.114	0.000	0.079	0.001
GroupActivities	Coefficient Tau	-0.063	0.161	0.046	-0.020	-0.082	0.125	-0.082	0.152	-0.102	0.183	-0.056	0.096
	P Value	0.008	0.000	0.509	0.771	0.001	0.000	0.013	0.000	0.005	0.000	0.096	0.004
HelpingOthers	Coefficient Tau	-0.034	0.114	0.155	-0.006	-0.037	0.126	-0.067	0.163	-0.025	0.127	0.137	0.064
	P Value	0.137	0.000	0.028	0.935	0.124	0.000	0.036	0.000	0.496	0.000	0.000	0.052
Home	Coefficient Tau	0.012	0.073	0.033	0.142	-0.023	0.061	-0.022	0.096	-0.034	0.121	0.005	0.139
	P Value	0.602	0.002	0.646	0.045	0.330	0.010	0.492	0.002	0.352	0.001	0.871	0.000
Interests	Coefficient Tau	0.024	0.057	-0.019	0.035	-0.043	0.086	-0.054	0.107	-0.090	0.091	-0.041	0.061
	P Value	0.311	0.014	0.787	0.622	0.075	0.000	0.091	0.001	0.013	0.011	0.219	0.066
Pet	Coefficient Tau	0.054	0.032	-0.037	0.110	0.007	0.031	0.029	-0.030	-0.040	0.044	0.041	-0.032
	P Value	0.025	0.182	0.611	0.128	0.775	0.203	0.373	0.359	0.282	0.231	0.230	0.341
Relaxation	Coefficient Tau	0.013	0.011	-0.059	0.162	-0.084	0.110	-0.104	0.103	-0.083	0.142	0.014	0.063
	P Value	0.585	0.636	0.403	0.020	0.000	0.000	0.001	0.001	0.022	0.000	0.686	0.059
SocialMedia	Coefficient Tau	0.053	-0.017	0.013	-0.123	0.067	-0.006	-0.010	-0.003	0.044	-0.049	0.103	0.006
	P Value	0.032	0.491	0.854	0.089	0.009	0.829	0.775	0.925	0.253	0.196	0.004	0.870
SpendingTime_Family	Coefficient Tau	-0.023	0.069	-0.034	0.125	0.019	0.074	0.030	0.034	-0.028	0.036	0.042	0.004
	P Value	0.331	0.003	0.629	0.073	0.433	0.002	0.350	0.290	0.442	0.310	0.214	0.901
SpendingTime_Others	Coefficient Tau	-0.018	0.098	-0.004	0.064	-0.064	0.138	-0.039	0.100	-0.075	0.096	-0.004	0.094
	P Value	0.425	0.000	0.953	0.351	0.007	0.000	0.214	0.001	0.038	0.007	0.903	0.005
Travelling	Coefficient Tau	0.027	0.029	0.047	-0.110	0.035	-0.014	0.076	-0.059	0.066	-0.055	0.018	-0.069
	P Value	0.273	0.235	0.525	0.136	0.170	0.586	0.025	0.075	0.082	0.143	0.614	0.046
WorkStudy	Coefficient Tau	0.020	0.082	-0.102	0.163	0.038	0.027	0.043	0.041	0.072	0.017	0.024	0.057
	P Value	0.393	0.001	0.149	0.020	0.128	0.272	0.187	0.212	0.052	0.640	0.476	0.093
WorldAffairs	Coefficient Tau	0.024	0.044	-0.029	0.023	-0.019	0.090	-0.062	0.062	-0.046	0.076	0.027	0.090
	P Value	0.340	0.074	0.693	0.753	0.450	0.000	0.067	0.063	0.228	0.042	0.439	0.010

Coefficient Tau and P Value broken down by phase (column), activity and PANAS catogory(i.e.positive, negative). The marks are labelled by Coefficient Tau and P Value.
Red: *p-value* < 0.05
Blue: *p-value* > 0.05
